# Case Report—Staged brachytherapy achieving complete metabolic response in unresectable oligometastatic colorectal cancer to the liver

**DOI:** 10.1093/omcr/omab016

**Published:** 2021-04-28

**Authors:** Gokula Kumar Appalanaido, Sheikh Izzat Bin Zainal-Abidin Bahajjaj, Syadwa Abdul Shukor, Muhammad Zabidi Ahmad, Ho Cho Hao Francis

**Affiliations:** 1 Department of Radiotherapy & Oncology, Advanced Medical & Dental Institute, Universiti Sains Malaysia, Penang, Malaysia; 2 Yong Loo Lin School of Medicine, National University of Singapore; 3 Department of Radiotherapy & Oncology, Sarawak General Hospital, Kuching, Malaysia; 4 Department of Radiology, Advanced Medical & Dental Institute, Universiti Sains Malaysia, Penang, Malaysia; 5 Department of Radiation Oncology, National University Cancer Institute Singapore

**Keywords:** Case Report, Brachytherapy, Liver Metastasis, Oligometastasis, Colorectal cancer

## Abstract

Liver is the most common site for metastasis from colorectal cancer (CRC). Non-surgical treatment options for oligometastatic CRC confined to the liver which represents an intermediate state in the metastatic cascade are fast expanding. Currently, several liver-directed local therapeutic options are available, such as hepatic arterial infusion (HAI) therapy, radio-frequency ablation (RFA), transarterial chemoembolization (TACE), stereotactic body radiotherapy and high dose rate brachytherapy (HDRBT). Many factors such as patient’s fitness, liver function (LF), tumour size, location of the tumour in the liver and scheduling of systemic therapy need to be considered when selecting patients for surgery or local liver-directed therapy. This case report illustrates a successful local treatment with staged HDRBT for a large and unresectable, liver only oligometastatic disease from CRC. This patient underwent 4 cycles of chemotherapy (FOLFOX 4) followed by primary tumour resection and first stage of HDRBT to liver for a residual 14 cm tumour after the chemotherapy. After completing a further 4 cycles of chemotherapy with the same regimen, the tumour remained stable at 8 cm. She underwent a second stage of HDRBT to the same lesion and a repeat PET-CT scan done 8 weeks after the second HDRBT showed complete metabolic response. To our knowledge, this is the largest CRC metastatic liver lesion that has been successfully treated with HDRB.

## INTRODUCTION

**Figure 1:**
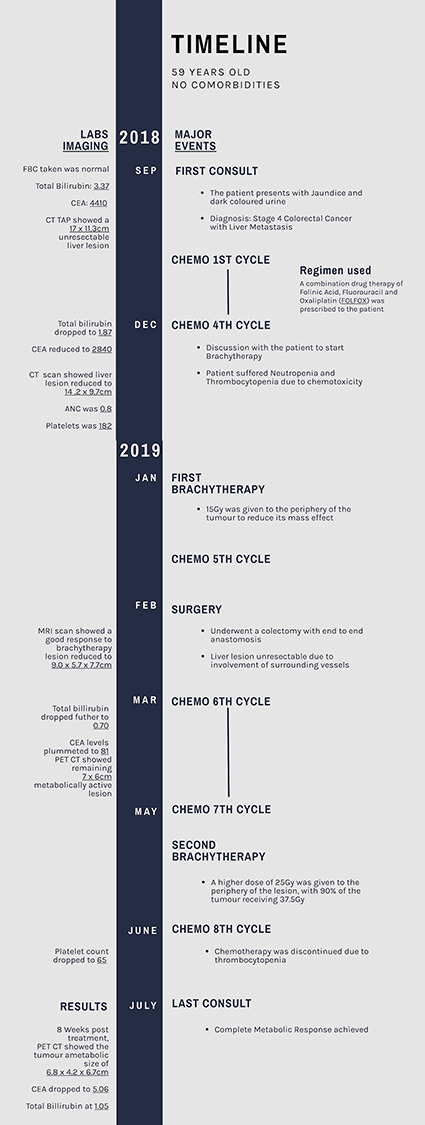
Timeline of Events.

It is estimated that 25-30% of patients with colorectal cancer develop liver metastasis, and around one third of them have oligometastatic liver disease [1]. For these patients, complete surgical resection is the treatment of choice. However, surgical resection is possible in less than 20% of patients [2], which sheds light on the importance of non-surgical treatment options. These options include radio-frequency ablation (RFA), trans-arterial chemoembolization (TACE), stereotactic body radiotherapy (SBRT) and high dose rate brachytherapy (HDRBT) [3]. We present a patient in which the strategy of pairing HDRBT sequentially with systemic chemotherapy resulted in the complete metabolic response of a large unresectable colorectal liver metastasis which is illustrated as a timeline (Fig. 1).

## CASE REPORT

A 59-year-old lady was diagnosed with Stage 4 Colorectal Adenocarcinoma with isolated Liver metastasis (T2N0M1). She was not a suitable candidate for surgery, and was referred for further oncological treatment.

Blood tests revealed significant liver impairment, with a Child’s Pugh Score of B8. Total Bilirubin (3.37 mg/dL), AST(130 U/L) and ALP (603 U/L) were elevated, while Albumin (34 g/L) was normal. As for her FBC, she had low Haemoglobin (10.5 g/dL), elevated White Cell Counts (12.3 x 10^9^/L), and normal platelet count (371 x 10^9^/L).

**Figure 2:**
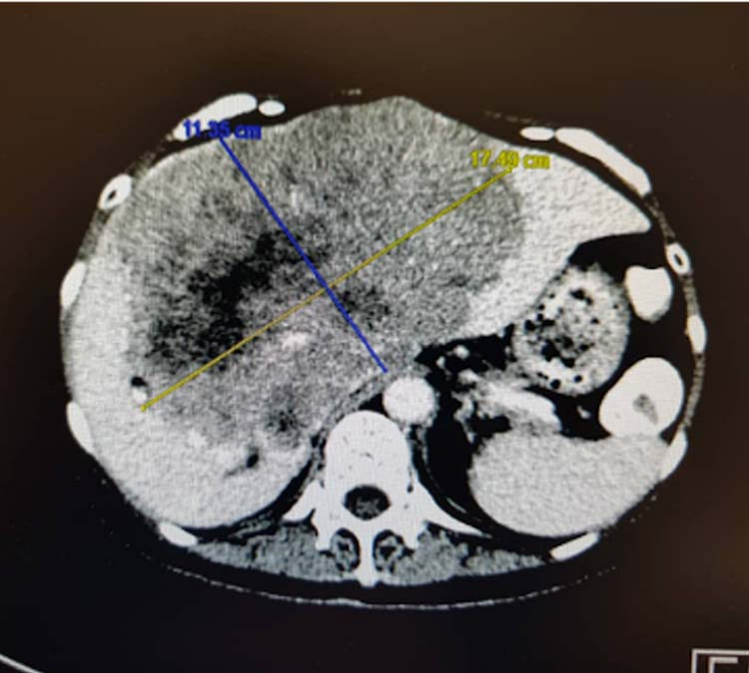
Axial CT scan showing liver metastasis at diagnosis.

A CT scan revealed a likely primary tumour at the proximal colon, a liver mass measuring 17.8 x 11.3 x 14.4 cm (Fig. 2), with elevated CEA at 4410 ng/mL. At the end of the entire course of treatment, CEA dropped to 5.06 ng/mL, suggesting that the liver metastasis originated from the sigmoid colon.

The patient was treated with a combination of liver HDRBT sandwiched between systemic chemotherapy.

**Figure 3:**
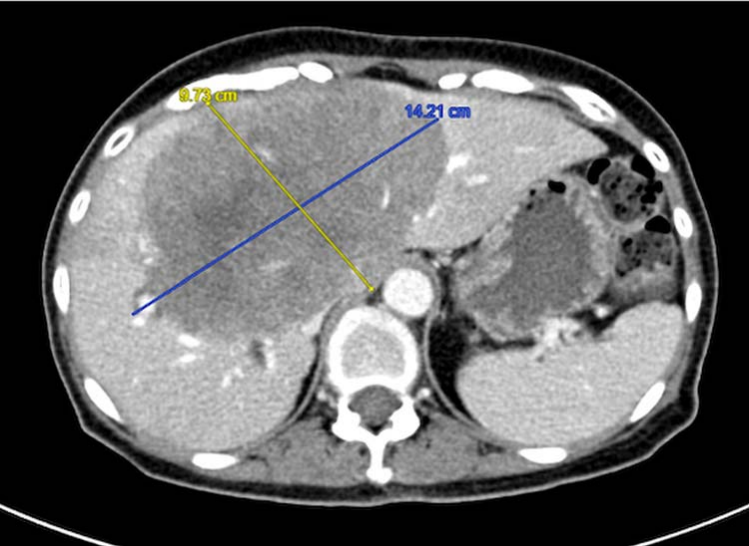
Axial CT scan demonstrating liver metastasis post 4 cycles of chemotherapy.

She underwent 4 cycles of systemic FOLFOX 4 chemotherapy and a CT scan afterwards showed that the liver lesion shrunk to 14.2 x 9.7 cm. (Fig. 3). Chemotherapy was however complicated by neutropenia and thrombocytopenia. She then consented to brachytherapy to control the liver lesion during this chemotherapy break, with the initial intention of shrinking the lesion as a bridge to surgical resection.

**Figure 4:**
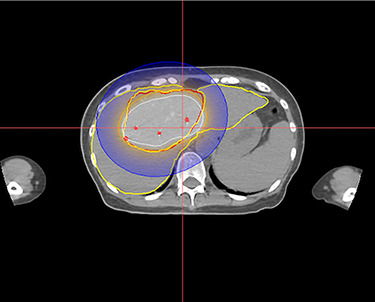
Stage 1 Brachytherapy Plan. Yellow contour—Liver. Red contour—tumour. White isodose line—20 Gy. Brown isodose line—15 Gy. Blue isodose line—5 Gy.

In January, the patient underwent her first Brachytherapy procedure. A dose of 15Gy was given to the periphery of the tumour (Fig. 4) via four brachytherapy applicators using Iridium 192 afterloader. Catheters were inserted using the anterior and right lateral approach. The applicators were placed in a way to adequately cover the entire tumour, with the first catheter inserted centrally within the tumour and the rest spaced out to cover the periphery of the tumour.

**Figure 5:**
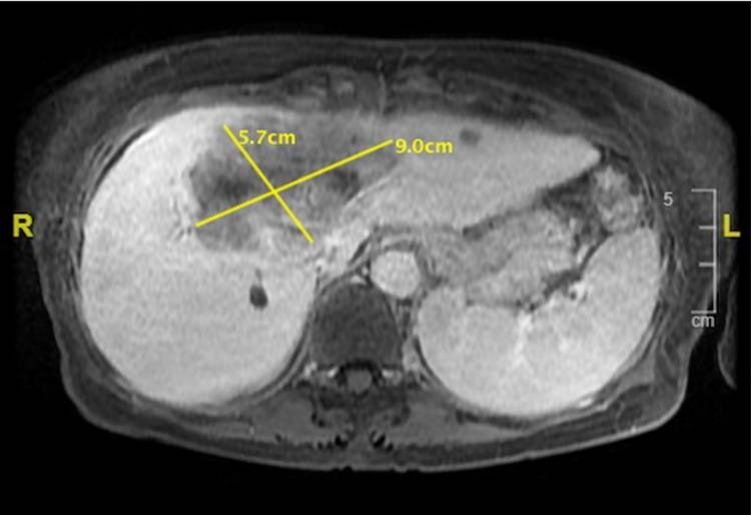
Axial MRI Liver demonstrating response to brachytherapy.

6 weeks later, an MRI scan showed good response with a 9.0 x 5.7 x 7.7 cm residual lesion. (Fig. 5).

The patient underwent further 4 cycles of chemotherapy. After the 5th cycle, she had a colectomy with end to end anastomosis to remove the primary tumour. Despite the reduced size of the liver lesion, the hepatobiliary surgeon deemed the mass unresectable due to the involvement of major vessels.

**Figure 6:**
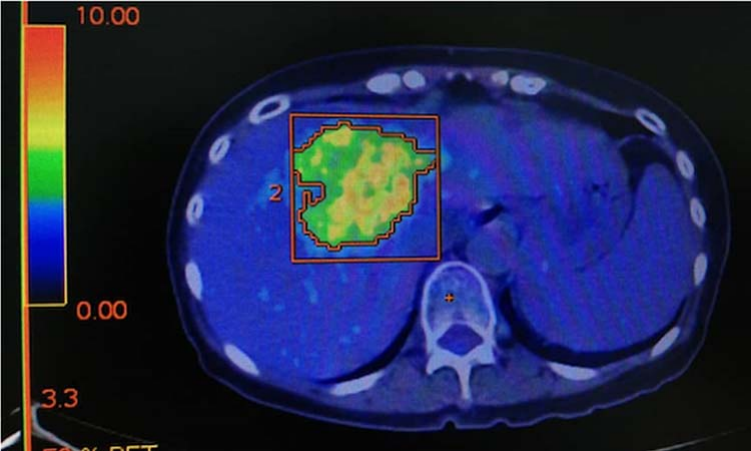
PET CT demonstrating residual liver tumour after 8 cycles of chemotherapy.

After 7 cycles, total bilirubin was 0.70 mg/dL and CEA dropped to 81. PET-CT scan still showed a remaining 7 x 6 cm metabolic active lesion with standardised uptake value (SUV) of 8.2. (Fig. 6). However, we were unable to complete the 8th cycle due to chemotherapy induced thrombocytopenia.

**Figure 7:**
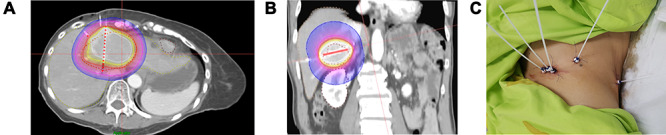
(A). Stage 2 Brachytherapy plan axial view. Dotted Yellow contour—Liver. Dotted Red contour—tumour. White isodose line—37.5 Gy. Red isodose line—25 Gy. Blue isodose line—12.5 Gy. (B). Stage 2 Brachytherapy plan coronal view. Dotted Yellow contour—Liver. Dotted Red contour—tumour. White isodose line—37.5 Gy. Red isodose line—25 Gy. Blue isodose line—12.5 Gy. (C). Clinical photo of interstitial catheters in situ.

She then underwent a second round of brachytherapy. A higher ablative dose of 25Gy was prescribed to the periphery of the lesion, with 90% of the lesion receiving 37.5Gy. (Fig. 7).

**Figure 8:**
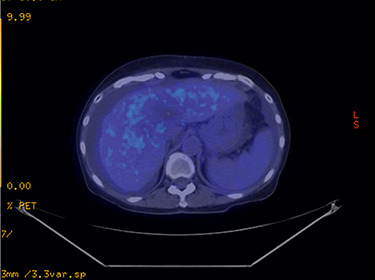
Axial PET CT demonstrating complete metabolic response post brachytherapy.

PET-CT scan after 8 weeks showed complete metabolic response (Fig. 8). There was an ametabolic tumour of 6.8 x 4.2 x 6.7 cm in size, SUV decreased from 8.1 to 4.8 units. Her total Bilirubin was 1.05 mg/dL, and CEA dropped to 5.06, but Albumin was noted to be at 28 g/L. On clinical examination, the patient was noted to have mild ascites.

## DISCUSSION

In this study, we delivered 2 staged HDRBT to the liver metastasis after the 4th cycle and 7th cycle of chemotherapy. Excellent clinical and biochemical results were achieved with a sharp fall in CEA from 4410 ng/ml to 5.06 ng/ml. The tumour regressed considerably with complete metabolic response. HDRBT liver has shown to be an excellent treatment option in this patient by inducing complete response in a very large unresectable tumour.

The first stage of brachytherapy with 15Gy after the initial 4 cycles of chemotherapy was to cytoreduce the tumour as it was too large for a curative dose. Liver size is not a major selection criterion provided that not more than 1/3 of normal liver receives more than 5Gy. RFA in contrast is not suitable due to the large tumour size and proximity to large vessels and bile ducts. Evidence shows that the main intra-hepatic bile ducts can withstand high doses of brachytherapy without significant toxicity unlike thermal heating [4].

Compared to SBRT, HDRBT has less issues with respiratory motion. A sharper dose fall off outside the target margin is another advantage of HDRBT in delivering tumoricidal doses [5]. HDRBT delivers a single large and precise dose to the tumour while sparring the surrounding normal liver tissues, increasing its therapeutic ratio. HDRBT is an ‘in-side out technique’ where extreme doses in multiplication of 2-3 times of the prescribed dose are delivered near the applicator, ideal for overcoming radioresistance due to tumour hypoxia. This is relevant as the large tumour predisposes it to tumour hypoxia in the core. Evidence on the use of HDRBT for liver tumours are growing in the literature and ESMO guidelines have recognized HDRBT liver as a local liver directed treatment option for CRC metastasis [6].

Generally HDRBT is a well-tolerated procedure. Fever within a few hours after the procedure is common. Minor complications like pain, nausea, vomiting, and asymptomatic pleural effusion 12 to 24 hours after treatment have been reported in about 10% of patients [7]. Rare major complications include liver abscess and subcapsular bleeding needing transfusion [8].

In conclusion, this approach of sandwiching HDRBT with systemic chemotherapy for large liver tumours needs to be further studied. This 2 stage approach of initial lower dose cytoreduction of tumour followed by an ablative dose of brachytherapy has proven to be a successful strategy in controlling the large liver metastasis in this case, that has not been previously described.

## Notes

Department and institution address where research was conducted: Department of Radiotherapy and Oncology, Advanced Medical & Dental Institute, Universiti Sains Malaysia, Bertam 13200 Kepala Batas, Pulau Pinang Malaysia
